# A prospective interventional trial on the effect of periodontal treatment on *Fusobacterium nucleatum* abundance in patients with colorectal tumours

**DOI:** 10.1038/s41598-021-03083-4

**Published:** 2021-12-09

**Authors:** Tsutomu Yoshihara, Mitomu Kioi, Junichi Baba, Haruki Usuda, Takaomi Kessoku, Michihiro Iwaki, Tomohiro Takatsu, Noboru Misawa, Keiichi Ashikari, Tetsuya Matsuura, Akiko Fuyuki, Hidenori Ohkubo, Mitsuharu Matsumoto, Koichiro Wada, Atsushi Nakajima, Takuma Higurashi

**Affiliations:** 1 Department of Gastroenterology and Hepatology, Yokohama City University School of Medicine, 3-9, Fukuura, Kanazawa-ku, Yokohama, Kanagawa 236-0004 Japan; 2 Department of Oral and Maxillofacial Surgery, Yokohama City University Graduate School of Medicine, Yokohama, Japan; 3 Department of Pharmacology, Faculty of Medicine, Shimane University, Izumo, Japan; 4 Department of Palliative Medicine, Yokohama City University Hospital, Yokohama, Japan; 5 Dairy Science and Technology Institute, Kyodo Milk Industry Co. Ltd, Tokyo, Japan

**Keywords:** Cancer, Microbiology, Gastroenterology

## Abstract

*Fusobacterium nucleatum* is associated with the progression of colorectal cancer. Thus, the possibility of preventing colorectal cancer or its progression by targeting *F. nucleatum* has been explored. As *F. nucleatum* is associated with periodontitis, we analysed whether treating periodontitis could influence *F. nucleatum* abundance in the colon. Patients with colorectal tumours who underwent colonoscopy were recruited. Patients diagnosed with periodontitis by a dentist were treated for approximately 3 months. Endoscopic resection of colorectal tumours was performed after periodontitis treatment, and resected tumours were pathologically classified as high-(HGD) or low-grade dysplasia (LGD). Saliva and stool samples were collected before and after the treatment. Of the 58 patients with colorectal tumours, 31 were included in the study, 16 showed improvement in periodontitis, and 11 showed no improvement. Stool *F. nucleatum* levels before treatment were significantly lower in the LGD group than in the HGD group. A significant decrease in faecal *F. nucleatum* levels was observed in patients who underwent successful treatment but not in those whose treatment failed. Salivary *F. nucleatum* levels were not altered in patients despite periodontal treatment. Thus, successful periodontitis treatment reduces stool *F. nucleatum* levels and may aid research on periodontitis and suppression of colorectal cancer development.

## Introduction

Colorectal cancer is the third most common malignant tumour in the world and is the second most common cause of cancer deaths^1^. The 5-year survival rate for colorectal cancer is approximately 10% for patients in advanced stages with metastasis and approximately 90% for those in the early stage. Therefore, early detection, treatment, and prevention are crucial for the recovery of patients^2^.

In 1982, John Robin Warren and Barry James Marshall discovered *Helicobacter pylori*, and its link to gastric cancer was extensively investigated^3^. *H. pylori* eradication significantly contributes to the prevention of gastric cancer. However, in the case of colorectal cancer, the microorganisms that are fundamental to carcinogenesis have not been fully identified. Using quantitative PCR, Castellarin et al*.* observed that *Fusobacterium nucleatum* was present in large numbers in colorectal cancer tissues, and that its presence was correlated with lymph node metastasis^4^. When compared with the bacterial flora in normal tissues of healthy subjects, the bacterial flora in normal colon tissues of colorectal cancer patients was more enriched in *F. nucleatum*; the diversity of intestinal bacteria in colon cancer tissues was reported to be lower than that in normal tissues located at a distance from cancer tissues^5^. According to a study that examined the amount of *F. nucleatum* DNA in colorectal cancer tissues by digital PCR, the amount of *F. nucleatum* DNA in colorectal cancer tissues was significantly higher than that in normal tissues. Additionally, the amount of *F. nucleatum* DNA tended to increase as the disease progressed or at an advanced stage of the disease^6^. Using conventional PCR, Mima et al*.* showed that patients with a high amount of *F. nucleatum* DNA in colorectal cancer tissues exhibit a poor prognosis^7^.

The National Health and Nutrition Examination Survey 2009–2014 reported that the incidence of periodontitis in the United States was 42.2% for all adults aged at least 30 years, and 7.8% for those with severe periodontitis^8^. Although daily oral care is considered important in the treatment of periodontitis, self-interruption of treatment by patients leads to its progression. Periodontitis has been considered a risk factor for systemic diseases such as diabetes, atherosclerosis, stroke, and fatty liver^9–12^. The development of these conditions may be attributed to an increase in the abundance of periodontitis-causing pathogens, which have been reported to cause hyper-endotoxemia^13^.

*F. nucleatum* is known to be associated with periodontitis. The predominance of oral bacteria in patients with periodontitis is altered by dysbiosis. Core species are bacteria whose proportions remain the same either under normal conditions or in periodontitis. *F. nucleatum*, one of the core species, activates the pathway of fermentation of lysine to butyrate at the affected periodontal sites. Butyrate levels are negatively correlated with oxygen levels, which are associated with the anaerobic conditions of periodontal pockets^14–16^. Therefore, *F. nucleatum* is one of the bacteria associated with inflammation in the oral cavity and is considered a causative agent of oral cancer^17^. *F. nucleatum* is thus considered to trigger carcinogenesis.

In our laboratory, we compared the strains of *F. nucleatum* in saliva and colorectal cancer tissues by arbitrarily primed PCR and identified the same strain of *F. nucleatum* in more than 40% of our subjects^18^. Analysing the strains is the first step towards identifying the *F. nucleatum* strains directly associated with colorectal cancer. It is plausible that colorectal cancer-associated *F. nucleatum* strains are derived from the oral cavity. Based on this study, we hypothesised that oral *F. nucleatum* and periodontitis are associated with colorectal cancer, and treatment of periodontitis would be an effective approach towards the prevention of colorectal cancer. The present study is a prospective, interventional trial, involving patients with colorectal tumours. We analysed the effect of periodontal treatment on *F. nucleatum* abundance in the stool and saliva of the subjects. In addition, changes in the gut microflora, occurring because of periodontal treatment, were analysed.

## Materials and methods

### Trial design and registration

This study was a prospective, interventional trial involving a single centre. It was conducted at Yokohama City University Hospital from August 2017 to August 2019. Clinical research was conducted in compliance with the regulations established by the Declaration of Helsinki. This study was approved by the Ethics Committee at Yokohama City University Hospital (B161201003) and was registered in the University Hospital Medical Information Network (UMIN) as UMIN000027352 on 16/05/2017. All patients enrolled in the study provided written informed consent.

The number of study subjects was approximately 30 because there are no studies on the extent to which *F. nucleatum* DNA levels are altered by periodontal treatment.

### Eligibility criteria

Patients with colorectal tumours who underwent colonoscopy were included. The age of the subjects ranged between 20 and 80 years. The following patients were excluded: patients who did not wish to undergo endoscopic treatment or periodontal treatment; those with advanced colorectal cancer (primarily because they require early therapeutic intervention); those consuming antibiotics or probiotics (because of their potential effect on the gut microbiota); and those with less than 10 teeth.

### Procedures

#### Periodontal treatments

The inclusion criteria for patients with periodontitis were as follows: patients who had not received any periodontal treatment within 6 months or any antibiotics within 3 months and had at least 10 residual teeth. The diagnosis of periodontitis was based on the JSP Clinical Practice Guideline for the Periodontal Treatment 2015 edited by The Japanese Society of Periodontology^19^. The depth of periodontal pockets (probing pocket depth; PPD) and the presence of bleeding during probing (bleeding on probing; BOP) were assessed. The periodontal examination sites and measurement methods were confirmed prior to the start of the study, and the examinations were performed by two designated dentists using a uniform method throughout the study period. For periodontal pockets, all the pockets were measured and the average value was recorded. Bleeding was recorded as the number of pockets that bled out of the total number of probed pockets. The severity of periodontitis in patients diagnosed based on the presence of attachment loss was classified according to the PPD values (mild: PPD < 4 mm; moderate: 4 mm ≤ PPD < 6 mm; severe: 6 mm ≤ PPD). After evaluation for periodontitis, oral hygiene instructions were provided to each patient followed by scaling and root planing procedures. Scaling was performed using a combination of ultrasonic and hand scalers, and root planing was performed in the subgingival area using ultrasonic and hand scalers under local anaesthesia. These procedures were conducted at the dental office at least once a month. Approximately three months after the first consultation and immediately before endoscopic resection of the colorectal tumours, the patients were evaluated for periodontitis. The ‘improvement group’ was defined as the one in which the post-treatment mean PPD was lower than the pre-treatment mean PPD and the post-treatment percentage of BOP was lower than or equal to the pre-treatment percentage. The ‘non-improvement group’ was defined as the one in which the post-treatment mean PPD was higher or equal to the pre-treatment mean PPD or the post-treatment percentage of BOP was higher than the pre-treatment percentage.

#### Colonoscopy and endoscopic tumour resection

The size and location of all tumours observed during colonoscopy were recorded. One of the colorectal tumours was biopsied, and a portion of the specimen was used for DNA extraction. Three months after colonoscopy, the tumour was endoscopically resected to the extent possible, and all resected tumours were evaluated by a pathologist. The tumour, identical to the colon tumour biopsied prior to periodontal treatment, was biopsied and used for DNA extraction. Tumours were classified into high-grade dysplasia (HGD) and low-grade dysplasia (LGD).

#### Collection of saliva and stool samples

Stool and saliva samples were collected before and after periodontal treatment. The patient was asked to gargle 10 mL saline for 1 min. The saliva specimen was centrifuged at 9100*g* for 3 min; the precipitate was stored at − 80 °C. Stool specimens were collected and stored at − 18 °C in a freezer at home. In our laboratory, they were stored at − 30 °C.

### Outcomes

The primary outcome measures included changes in the amount of *F. nucleatum* DNA in stool and saliva samples and colorectal tumours. We analysed the difference in the amount of *F. nucleatum* DNA in different histological tissue types before periodontal treatment. The secondary outcome measures included changes in the gut microbiota in stool and saliva.

### DNA extraction

Stool samples: DNA was extracted according to a published protocol with minor modifications^20^. Twenty milligram of faeces was suspended in 1 mL PBS and centrifuged three times at 14000*g* for 5 min. Extraction buffer (450 µL) (100 mM Tris–HCl, 40 mM EDTA, pH 9.0) was used to resuspend the samples. Next, 50 µL 10% sodium dodecyl sulphate, 300 mg glass beads (0.1 mm in diameter) (TOMY, Tokyo, Japan), and 500 µL buffer-saturated phenol were added to the samples. Micro Smash (4000 rpm, 10 s) (TOMY, Tokyo, Japan) was used for cell disruption, and the cells were heated at 65 °C for 10 min. Cell disruption and heating were repeated. The solution was centrifuged at 20,000*g* for 10 min, and 400 µL phenol–chloroform-isoamyl alcohol (25:24:1) was added to 400 µL of the supernatant, which was resuspended and centrifuged at 2000*g* for 10 min. To 250 µL of the supernatant, 25 µL 3 M sodium acetate (pH 5.2) and 250 µL ice-cold isopropanol were added. The suspension was centrifuged at 20,000*g* for 15 min. The supernatant was removed and centrifuged at 20,000*g* for 5 min with 500 µL 70% ethanol. The supernatant was removed and dried, and 1 mL TE (10 mM Tris–HCl 1 mM EDTA, pH 8.0) was used to dissolve the extracted DNA.

Saliva samples: Bacterial DNA was extracted from the saliva by using a NucleoSpin® DNA Stool kit (MACHEREY–NAGEL GmbH & Co. KG, Dueren, Germany) according to the manufacturer's instructions.

Colorectal tumour samples: DNA was extracted from tumour samples by using the QIA amp DNA Mini Kit (Qiagen, Venlo, The Netherlands) according to the manufacturer's instructions.

### Bacterial flora analysis

DNA extraction was performed as previously described; the obtained DNA was stored at − 80 °C until further use. Analysis of the V3–V4 region of bacterial 16S rRNA was performed using a published protocol with minor modifications^21–23^. Briefly, the amplicons containing the V3-V4 region of 16S rRNA and unique indices incorporated by an Illumina Nextera XT Index kit v2 (Illumina. K., Japan) were purified using AMPure XP beads (Beckman Coulter, Inc., Brea, CA, USA). The purified barcoded DNA library was diluted to 4 nmol/L using 10 mmol/L Tris–HCl (pH 8.0), and the same volume of each sample was pooled for multiplex sequencing. The multiplexed library pool (6 pmol/L) was spiked with 5% PhiX control DNA (6 pmol/L) and was sequenced using a 2 × 300 bp paired-end run on a MiSeq platform using a MiSeq Reagent Kit v3 (Illumina). All quality-approved, trimmed, and filtered sequences were processed using a custom script based on the QIIME software suite (http://qiime.org/)^24^.

Diversity analysis: Alpha diversity was applied to analyse the complexity and species diversity of samples via two indexes: Chao1 and Shannon index. These indices were calculated with QIIME and displayed with the package ‘diversity’ in the R software (Version 3.6.1). Beta-diversity analysis was used to evaluate the differences in species diversity of samples. To calculate the beta-diversity values, cluster analysis was preceded by principal coordinate analysis (PCoA) using the R Software Version 3.6.1 (https://www.r-project.org/).

### Digital PCR

The extracted DNA was diluted with RNAase-free water. The reaction mixture included 33 ng of DNA, RNAase-free water, 7.5 µL QuantStudio™ 3D Digital PCR Master Mix v2 (Applied Biosystems), and primers (made up to a volume of 14.5 µL) and was applied to the QuantStudio™ 3D Digital PCR 20 K Chip Kit v2 (Applied Biosystems). The primer sequences are as follows: forward primer, 5′-AAGCGCGTCTAGGTGGTTATGT-3′; reverse primer, 5′-TGTAGTTCCGCTTACCTCTCCAG-3′; and probe, 5′-FAM-CACGCAATACAGTTGAGCCCTGCATT-3′ (Applied Biosystems). The ProFlex PCR System (Applied Biosystems) thermocycler was used for amplification. DNA was amplified by initial denaturation at 96 °C for 10 min, followed by 39 cycles of 56 °C for 2 min, 98 °C for 30 s, 60 °C for 2 min, and a final hold at 10 °C. The chips were read using the QuantStudio™ 3D Digital PCR instrument (Applied Biosystems) and analysed using the QuantStudio 3D Analysis Suite Software (Applied Biosystems).

### Statistical analysis

All statistical analyses were performed using JMP^®^ 15 (SAS Institute Inc., Cary, NC, USA). Data are shown as means ± standard error. A paired Student’s *t*-test was performed to compare the two groups before and after treatment (Figs. 2, 3b,d, 4a,c, S1b, and S2). Shapiro–Wilk's test was used to determine if the data followed a normal distribution; the Wilcoxon rank-sum test was used for data types that followed a non-normal distribution (Figs. 3a and S1a). Fisher’s exact tests were performed for categorical data (Table 1). A p-value less than 0.05 was considered significant. For the gut microbiome analysis, we calculated the false discovery rate (FDR) using the Benjamini and Hochberg method. An FDR value of less than 0.1 was defined as statistically significant.

**Table 1:** Clinical characteristics of patients.

	Improvement group (n = 16)	Non-improvement group (n = 11)	p-value
Age, mean (SD)	66.8 (11.0)	68.4 (10.0)	0.75
Gender (M:F)	11:5	7:4	1
BMI, mean (SD)	24.3 (3.7)	25.9 (2.1)	0.20
Ratio of HGD	0.25	0.45	0.41
Number of tumours (SD)	3.6 (2.2)	5.5 (4.7)	0.36
**Ratio of complications**			
Diabetes	0.25	0.27	1
Hypertension	0.44	0.64	0.44
Dyslipidemia	0.38	0.36	1
Cardiovascular disease	0.13	0	0.50
**Periodontitis grade**			
Mild	2	4	0.42
Moderate	6	3	
Severe	8	4	

## Results

### Study flow and patient characteristics

**Figure 1:**
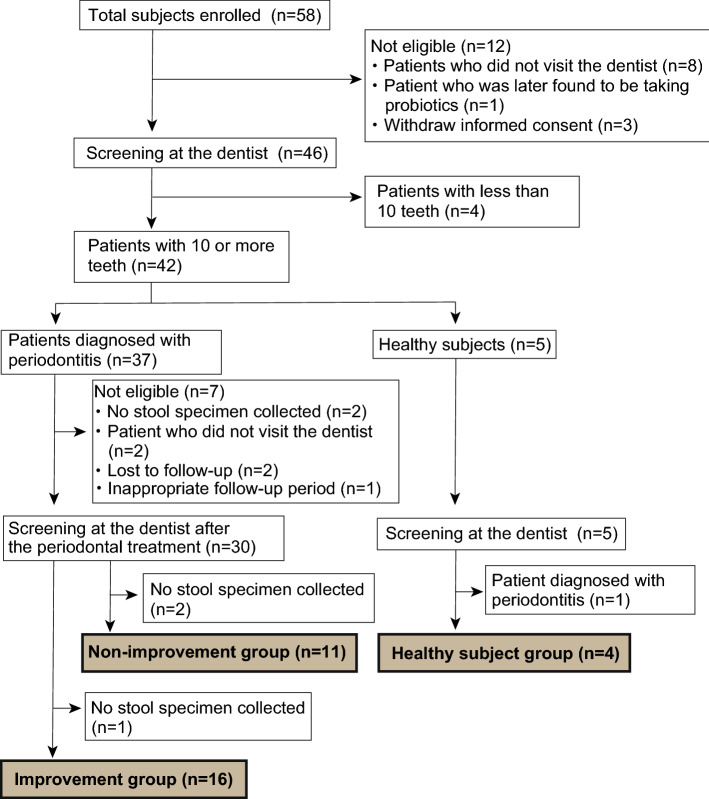
Flow diagram of the study.

Written informed consent was obtained from 58 patients who underwent colonoscopy and were diagnosed with colorectal tumours. Thirty-one patients were evaluated as the following groups: improvement (n = 16), non-improvement (n = 11), and healthy subjects (n = 4). Twenty patients were excluded after participation in the study: eight patients did not visit the dentist; one patient was found to be consuming probiotics, and three patients withdrew their consent. Four patients were excluded after the screening by the dentist because they had less than 10 teeth remaining. Of the 37 patients diagnosed with periodontitis, seven patients were excluded: two patients whose stool specimens were not collected, two patients who did not visit the dentist, two patients who did not appear for follow-up consultations, and one patient with an inappropriate follow-up period. Of the 30 patients who were examined by a dentist after periodontal treatment, two patients in the improvement group and one patient in the non-improvement group were excluded because stool specimens could not be collected from them. In the healthy subject group, one patient who showed periodontitis after 3 months of screening was excluded (Fig. 1). The clinical characteristics of patients are shown in Table 1.

### Changes in the mean value of PPD and the percentage of BOP after treatment of periodontitis

**Figure 2:**
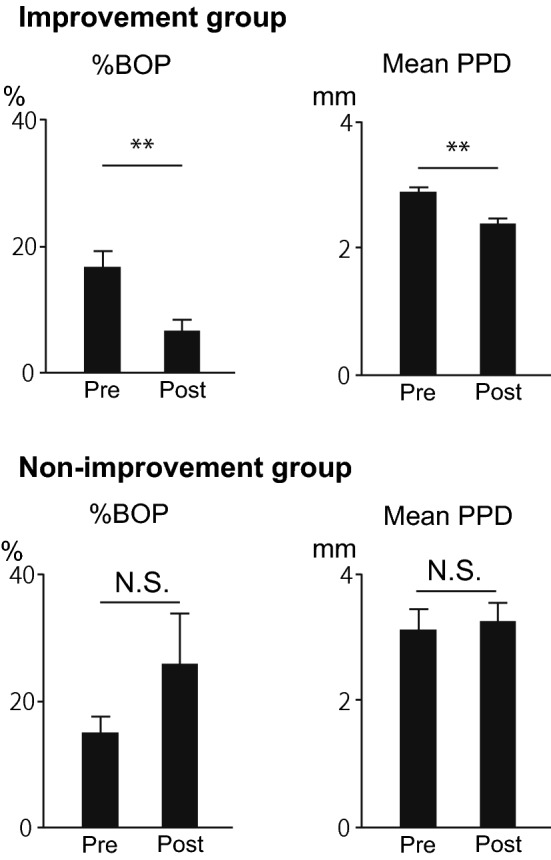
Results of periodontal treatment in patients. The %BOP is the percentage of periodontal pockets that bled when probed out of the total periodontal pockets. In the improvement group, both %BOP and mean PPD improved with periodontal treatment (n = 16, 11). Data are means ± SEM. **p < 0.01, paired Student’s *t*-test. BOP, bleeding on probing; PPD, probing pocket depth.

Although PPD values appeared low even in the presence of periodontitis, we calculated the average value of PPD for all pockets, which resulted in a low average value of PPD. The mean value of PPD and the percentage of BOP in patients significantly decreased after treatment (p < 0.01, p < 0.01, respectively) in the improvement group. In the non-improvement group, the mean value of PPD and the percentage of BOP were not significantly different after treatment (Fig. 2).

### *F. nucleatum* DNA levels in stool and tumours before periodontal treatment

**Figure 3:**
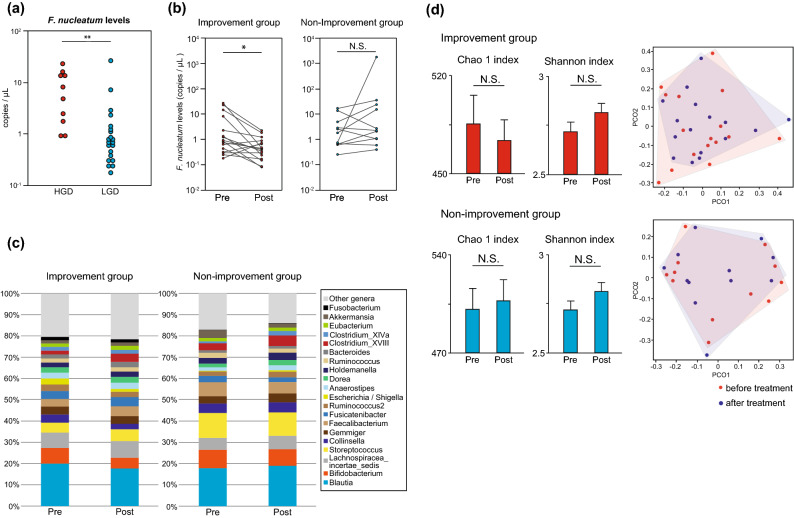
Analysis of *Fusobacterium nucleatum* DNA levels and bacterial flora in the stool. (**a**) *F. nucleatum* DNA levels in the stool before periodontal treatment (n = 10, 21). HGD, high-grade dysplasia; LGD, low-grade dysplasia. (**b**) *F. nucleatum* DNA levels in the stool of patients before and after periodontal treatment (n = 16, 11). (**c**) Changes in the bacterial composition of the stool before and after treatment for periodontitis. (**d**) Changes in the diversity of faecal bacterial flora. Data are means ± SEM. *p < 0.05, **p < 0.01, Wilcoxon rank-sum test (**a**), paired Student’s *t*-test (**b**,**d**), PCoA analysis with the R Software (**d**).

*F. nucleatum* DNA levels in stool were measured separately for patients with HGD and patients with only LGD (no HGD, LGD group). Figure 3a shows a comparison of *F. nucleatum* DNA levels in stool between the LGD and HGD groups; the LGD group showed significantly lower amounts of *F. nucleatum* DNA in their stool (p < 0.001) (Fig. 3a). *F. nucleatum* DNA levels in colorectal tumours were measured before and after periodontal treatment, but no significant changes were observed in either group (Supplementary Fig. S1).

### Changes in *F. nucleatum* DNA levels in stool samples and gut microflora analysis before and after periodontal treatment

*F. nucleatum* DNA levels in stool before and after periodontal treatment were quantified by digital PCR (Fig. 3b). In the improvement group, the amount of *F. nucleatum* DNA was significantly decreased in the stool of patients after treatment (p < 0.05). However, in the non-improvement group, there was no significant change in *F. nucleatum* DNA levels before and after treatment; in fact, several samples showed an increase in *F. nucleatum* DNA amount. As the amount of *F. nucleatum* DNA in stool is associated with the status of periodontal treatment, it may serve as a sensitive marker for the success or failure of the treatment. Furthermore, next-generation sequencing of stool samples revealed no significant changes in bacterial composition before and after treatment (Fig. 3c). There were no significant changes in the Chao1 index and Shannon index before and after treatment. According to PCoA, no clustering shifts occurred before and after treatment (Fig. 3d). These results indicated that periodontal treatment did not change the bacterial composition of the stool.

### Changes in *F. nucleatum* abundance and analysis of salivary microflora before and after treatment of periodontitis

**Figure 4:**
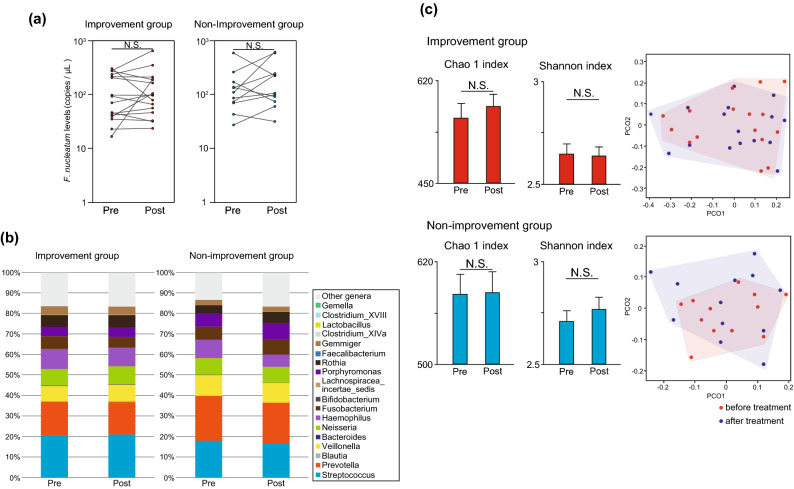
Analysis of *Fusobacterium nucleatum* DNA levels and bacterial flora in saliva. (**a**) *F. nucleatum* DNA levels in the saliva before and after periodontal treatment (n = 16, 11). (**b**) Changes in the bacterial composition of saliva before and after treatment for periodontitis. (**c**) Changes in the diversity of the bacterial flora in saliva. Data are means ± SEM. Paired Student’s *t*-test, PCoA analysis with the R Software (**c**).

The bacterial flora was analysed using DNA extracted from the saliva of patients before and after periodontal treatment. Digital PCR analysis showed no difference in the amount of *F. nucleatum* DNA before and after treatment in both the improvement and non-improvement groups (Fig. 4a). No significant change in bacterial flora was observed before and after treatment in both the groups (Fig. 4b). There were no significant changes in Chao1 and Shannon indices before and after treatment. PCoA revealed that no clustering shift occurred (Fig. 4c). It was suggested that the periodontal treatment that we performed may not be able to alter the salivary flora and *F. nucleatum* abundance and that changes in the microbial flora and *F. nucleatum* abundance in saliva may not be directly linked to a decrease in *F. nucleatum* abundance in the stool.

## Discussion

In this study, we show that successful periodontal treatment could reduce *F. nucleatum* abundance in the stool. In contrast, there was no decrease in the abundance of *F. nucleatum* in the saliva and no significant change in the gut microbiota despite periodontal treatment.

According to the guidelines of the European Federation of Periodontology, the endpoint of intervention is improvement in PPD and BOP^25^. In addition, it has been reported that the bacterial load of periodontal pathogens, including *F. nucleatum*, is correlated to PPD and BOP^26^. Therefore, we considered it appropriate to use PPD and BOP as indicators of periodontal therapy. In several patients in this study, the treatment for periodontitis was unsuccessful. The treatment of periodontitis included voluntary routine oral care by the patients. Two factors prevented many cases of periodontitis from improving: (1) the short period of time (3 months) between the initiation of treatment and the resection of the colorectal tumour, and (2) oral self-care by the patient in addition to professional care. However, if periodontitis is successfully treated, it is possible to reduce *F. nucleatum* abundance in the stool.

Yachida et al*.* showed that *F. nucleatum* abundance increased with the progression of colorectal cancer and that *Atopobium parvulum* and *Actinomyces odontolyticus* abundance increased during the carcinogenesis stage and decreased as the disease progressed. This finding suggests that *A. parvulum* and *A. odontolyticus* may play a role in the mechanism of carcinogenesis of colorectal cancer and that *F. nucleatum* is associated with its progression^27^. PCR analysis revealed a higher level of *F. nucleatum* DNA in tissues from patients with colorectal cancer and those with high-grade adenomas than in the control, but there was no significant increase in the amount of *F. nucleatum* DNA in tissues from patients with tubulovillous adenomas or low-grade adenomas^28^. This suggests that *F. nucleatum* may be associated with the initiation of cancer. It has been reported that Fap2 and Fad-A, which are expressed in *F. nucleatum*, are involved in the growth of colorectal cancer and inflammatory responses^29,30^. Fap2 of *F. nucleatum* binds to Gal-Gal-NAc, which is overexpressed in colorectal cancer tissues. Fad-A proteins expressed in *F. nucleatum* adhere to cells and invade cells via E-cadherin, a cell adhesion molecule. Fad-A promotes the growth of colorectal cancer cells by activating β-catenin signalling and promoting the expression of *Wnt* genes and oncogenes. Therefore, reducing the population of *F. nucleatum* in the colon may contribute to suppressing the progression or initiation of colorectal cancer. In the current study, *F. nucleatum* levels in colorectal tumours were not significantly changed upon periodontitis treatment. There were several patients with LGD in this study, and there may be a weak correlation between the presence of *F. nucleatum* and tumour tissues in LGDs. A larger number of HGD tissues may be analysed in future studies to determine whether periodontal treatment influences *F. nucleatum* levels in tumours.

In the present study, notably, *F. nucleatum* abundance in the stool of patients with LGDs was low. Flanagan et al. reported that faecal *F. nucleatum* abundance was higher in patients with HGD than in patients with LGD and control patients, and there was no relationship between the amount of *F. nucleatum* DNA in stool and that in tumour tissue samples from the same patients^28^. Amitay et al*.* reported that *F. nucleatum* abundance in the stool of patients with colorectal cancer was significantly higher than that in patients with no signs of neoplasm, non-advanced adenoma, and advanced adenoma^31^. We used digital PCR because of its high sensitivity, which makes it a more suitable tool for the quantification of *F. nucleatum*. Cases of LGDs are frequently detected and the amount of *F. nucleatum* DNA in the stool of patients with LGD is often low^28^. Digital PCR has the advantage of detecting target DNA with high sensitivity and provides absolute quantification. In a report on the detection of *Salmonella typhimurium* using both digital PCR and real-time PCR, digital PCR detected lower amounts of DNA and functioned in the presence of inhibitors^32^.

*F. nucleatum* abundance and bacterial composition of the saliva of patients remained unaltered in the present study, similar to observations in previous studies^33,34^. Although the bacterial composition of saliva did not change after periodontal treatment, the abundance of *Fusobacterium* in the supragingival plaque has been reported to decrease after treatment^33^. It is possible that changes occurred in subgingival or supragingival plaques; however, this was not analysed in the present study because our previous research led us to believe that *F. nucleatum* is transferred to the colon by the swallowing of saliva^18^. It has been reported that the salivary flora has a circadian rhythm with a 24-h cycle^35^. In our study, the saliva was collected immediately before the endoscopies to avoid a large difference between the composition at the starting time of the first and second endoscopies. Nevertheless, the time was not exactly the same due to the time required to finish bowel preparation. It is possible that this is the reason why no changes in salivary *F. nucleatum* levels were observed. If *F. nucleatum* is transferred to the colon via the gastrointestinal tract and the amount of *F. nucleatum* swallowed per day was altered by periodontal treatment, it could have been observed as a change in *F. nucleatum* in the stool.

Several patients with ulcerative colitis and Crohn's disease also exhibit periodontitis^36^. *F. nucleatum* is abundantly detected in the colonic mucosa of patients with ulcerative colitis and has been reported to affect the activity and clinical course of ulcerative colitis, promote mucosal injury, and increase the expression of inflammatory cytokines^37^. In addition, inflammatory bowel disease is influenced by pro-inflammatory cytokines in the gingiva^38^, and improvement in the periodontitis status may alleviate inflammation in the colon. It is possible that *F. nucleatum* levels decrease as colonic inflammation improves, or vice versa. It is also plausible that changes in the bacterial flora of the gingival plaque affect the colon. According to a previous study, the abundance of *F. nucleatum* in supragingival plaques decreases after periodontal treatment^33^, and this reduction may be associated with a decrease in *F. nucleatum* abundance in the stool. In a previous study, blood samples obtained from patients with periodontitis after brushing were analysed by PCR. The results revealed a higher incidence of bacteraemia in these patients^39^. In a mouse model of colorectal cancer, *F. nucleatum* was injected into the tail vein of mice. *F. nucleatum* localised to mouse tumour tissues in large numbers via Fap2, indicating that *F. nucleatum* reached colorectal tumour tissues via a haematogenous route^29^. These findings suggest that brushing may cause dissemination of *F. nucleatum* into the bloodstream, allowing it to access and bind to colorectal cancer tissues. If *F. nucleatum* is transferred to the colon via the bloodstream from periodontal pockets, it is possible that periodontal intervention decreased subgingival and colonic *F. nucleatum* abundance. In addition, biofilms produced by oral bacteria influence colon health and cause chronic inflammation, which is thought to be one of the mechanisms underlying the development of colorectal cancer^40^. The effect of periodontal treatment on the bacterial composition of saliva remains limited, as the composition is strongly influenced by the entire upper respiratory tract, including the pharynx, and not the oral cavity alone.

The limitation of this study is that the subjects did not include patients in advanced stages of colorectal cancer. *F. nucleatum* abundance in the stool of patients included in this study was lower than that observed in patients with advanced cancer^28^. The benefit of periodontal treatment for patients with colorectal cancer in the advanced stages could not be examined. Additionally, plaque analysis could not be performed because oral plaque samples were not collected. Furthermore, although the amount of *F. nucleatum* DNA in colorectal tumours did not change significantly before and after periodontal treatment, the possibility of contamination by stool adhering to the tumour tissue surface could not be ruled out. Thus, accurate analyses may not have been possible. Finally, this study is not a randomised controlled trial; it is an exploratory trial, because no previous studies have reported the extent to which *F. nucleatum* DNA levels are altered by periodontal treatment. Therefore, the number of subjects was low. Additionally, the healthy subject sample size was small because we intended to focus our analysis on patients who underwent periodontal interventions.

In summary, we show that periodontal treatment reduces *F. nucleatum* abundance in the stool of patients with colorectal tumours. Although it is unclear whether the reduction in *F. nucleatum* abundance in stool can prevent the initiation and progression of colorectal cancer, we believe that this study contributes to the development of research on the possible effects of periodontal treatment on colorectal cancer. Additional studies are needed to verify the mechanism by which *F. nucleatum* levels in the stool are reduced.

## Supplementary Information

Supplementary Information 1.

Supplementary Information 2.

## Data Availability

The data that support the findings of this study are available from the corresponding author, upon reasonable request.
