# Supplementary material for: A prospective interventional trial on the effect of periodontal treatment on Fusobacterium nucleatum abundance in patients with colorectal tumours
Source: Sci Rep. 2021 Dec 9;11:23719. doi: 10.1038/s41598-021-03083-4 (PMC8660914; doi:10.1038/s41598-021-03083-4)
Supplement: Supplementary file 1 — Supplementary Information 1. [file 41598_2021_3083_MOESM1_ESM.pdf]

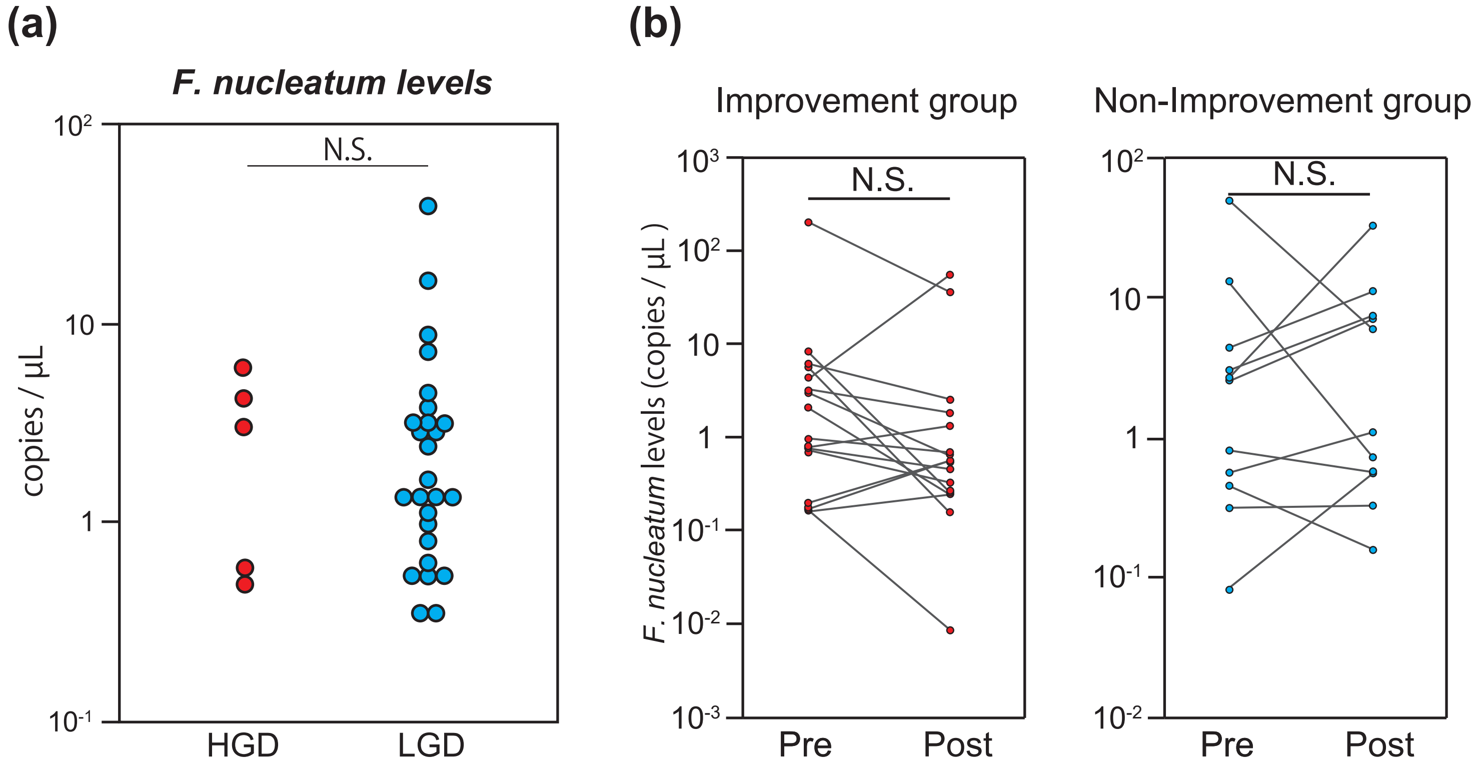

Figure S1. Analysis of *F. nucleatum* DNA levels in colorectal tumours. (a) *F. nulcleatum* DNA levels in colorectal tumours before periodontal treatment (n=5, 26). (b) *F. nucleatum* DNA levels in colorectal tumours before and after periodontal treatment (n=16, 11). Wilcoxon rank-sum test (a), paired Student's t-test (b).
