# Supplementary material for: A prospective interventional trial on the effect of periodontal treatment on Fusobacterium nucleatum abundance in patients with colorectal tumours
Source: Sci Rep. 2021 Dec 9;11:23719. doi: 10.1038/s41598-021-03083-4 (PMC8660914; doi:10.1038/s41598-021-03083-4)
Supplement: Supplementary file 2 — Supplementary Information 2. [file 41598_2021_3083_MOESM2_ESM.pdf]

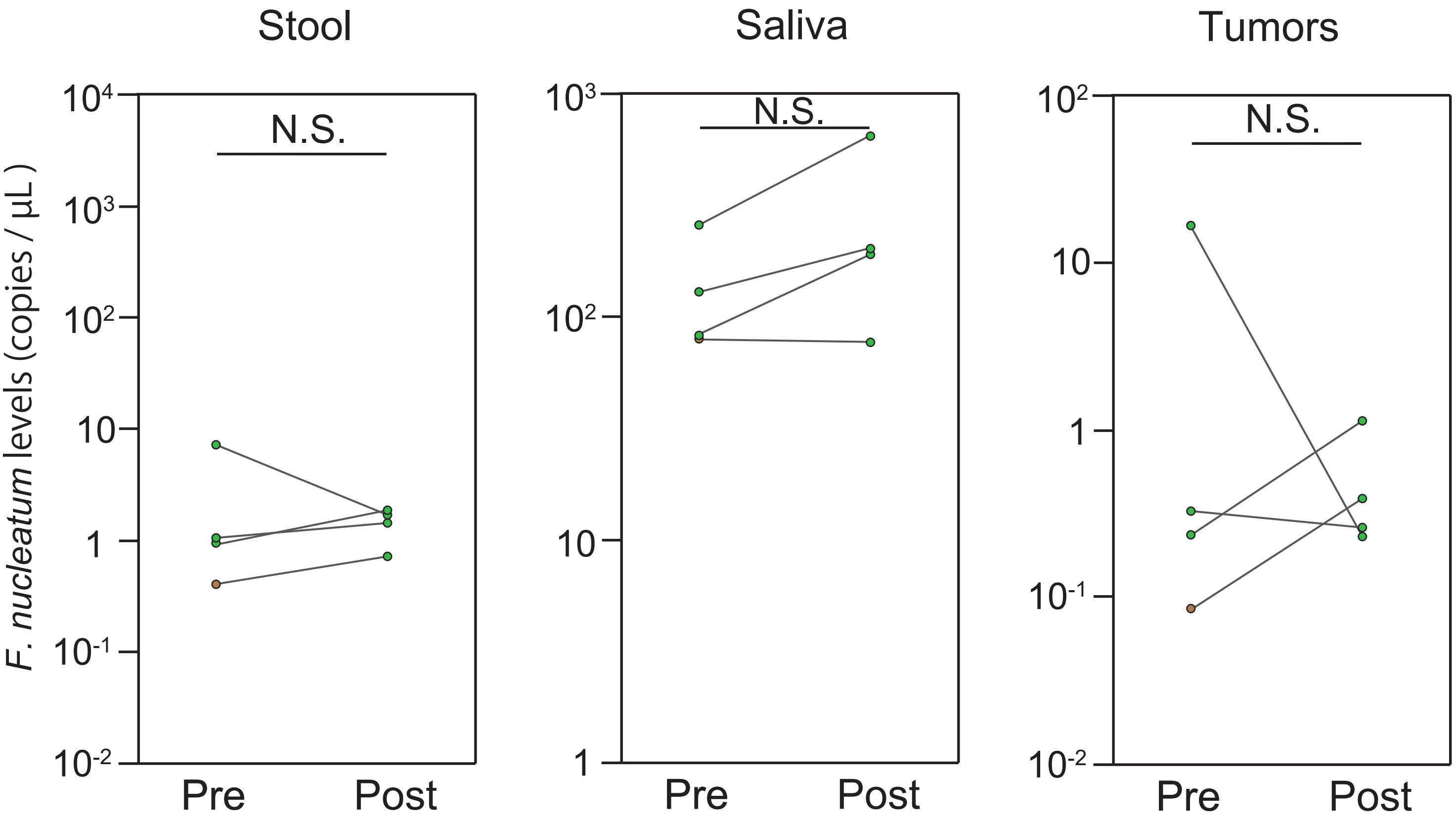

Figure S2. *F. nucleatum* DNA levels before and after periodontal treatment in the healthy subject group (n=4). Paired Student's t-test.
